# Parental knowledge and usage of air quality in childhood asthma management

**DOI:** 10.3389/fped.2022.966372

**Published:** 2022-10-26

**Authors:** Jessica Reyes-Angel, Yueh-Ying Han, Erick Forno, Juan C Celedón, Franziska J Rosser

**Affiliations:** Department of Pediatrics, Division of Pulmonary Medicine, UPMC Children's Hospital of Pittsburgh, University of Pittsburgh, Pittsburgh, PA, United States

**Keywords:** childhood asthma, air quality index, air pollution, parental knowledge and practice, air quality index (AQI)

## Abstract

**Background:**

The current United States asthma management guidelines recommend usage of the Air Quality Index (AQI) for outdoor activity modification when air pollution is high. Little is known about parental knowledge and usage of air quality including the AQI in managing childhood asthma.

**Methods:**

Forty parents (or legal guardians) of children with persistent asthma completed a questionnaire designed to assess 4 areas related to outdoor air pollution: awareness, perception, behavioral modification, and prior healthcare provider discussion. Descriptive statistics were obtained and Fisher's exact test was used for analysis of behavioral change by selected variables.

**Results:**

Almost all parents reported awareness of air quality alerts or AQI, however, only 20% checked the AQI on the AirNow app or website. Most parents reported air pollution as a trigger (65%), yet few parents reported behavioral modification of their child's outdoor activity based on the perception of poor air quality (43%) or based on AQI or alerts (40%). Over half of parents reported a healthcare provider had ever discussed air pollution as a trigger, with few parents (23%) reporting recommendations for behavior change. Perception of air pollution as a trigger, healthcare provider discussion and recommendations, and usage of AirNow were associated with increased reported activity change.

**Conclusion:**

Healthcare providers should discuss outdoor air pollution during asthma management in children and should discuss AirNow as a source for AQI information and behavioral recommendations.

## Introduction

Outdoor air pollution is a well-known cause of childhood asthma exacerbations (1). Current United States (US) asthma management guidelines (2) and the American Academy of Pediatrics (3) advise usage of the Air Quality Index (AQI) to reduce outdoor air pollution exposure. While recommending usage of the AQI is prudent, little is known about the usage or efficacy of personal exposure avoidance strategies, and health outcomes (4). Further, there is little data to guide parents and children with asthma on how the AQI should be used. For example, the AQI as a tool for behavioral change can be utilized in two ways: by responding to AQI alerts which are generally issued for days forecast to have air pollution above the National Ambient Air Quality Standard (NAAQS) or by checking the AQI prior to outdoor activity on the AirNow website or app as a way to learn individual susceptibility to air pollution. Despite national recommendations, it remains largely unknown if parents of children with asthma are aware of outdoor air pollution risks or the AQI, whether and how they incorporate air pollution avoidance strategies in their child's asthma management, or whether they discuss such strategies with health care professionals (5).

To date there has been one prior publication examining parents of children with asthma knowledge and response to air quality alerts (6). To our knowledge, no study has evaluated parental knowledge and usage of the AirNow app. Our objective was to characterize parental knowledge and usage of the AQI in their child's asthma management in a convenience sample of 40 parents.

## Materials and methods

An 11-item questionnaire was developed to assess parental air quality knowledge, perceptions, behavioral change, and discussion with health professionals including adapting questions used in the National Health and Nutritional Examination Survey (NHANES) (7), Behavioral Risk Factors Surveillance Survey (8), and prior AQI publications (9) (Supplementary Materials Table S1). We evaluated parental reported change in two ways: based on perception of poor air quality, which may differ from true air quality (10, 11), and based on the AQI or alerts. Parents or legal guardians (collectively referred to as “parents”) of children aged 8–17 years with physician diagnosed persistent asthma were eligible to receive the questionnaire. Parents were eligible to participate if their child was participating in a separate study (NCT: 04454125), which recruited children from a pediatric pulmonology clinic and asthma registry during July- October 2020. For the separate study, the older ages of children were selected for the ability to check the AQI and self-regulate activity. All eligible parents participated. The questionnaire was administered to the parents after written informed consent was obtained. The study was approved by the University of Pittsburgh Institutional Review Board.

Descriptive statistics were obtained for select parental and child demographics and for questionnaire data. Race and sex of both parent and child were self-reported. Questionnaire response choices of “Don”t Know/ Not Sure” and “Never thought about it” were coded as negative (9, 12). Fisher's exact test was used for comparison between groups. All analyses were conducted using SAS v 9.4 (SAS Institute Inc). Two-sided *P* values less than .05 were considered statistically significant.

## Results

Table 1 displays characteristics of the 40 enrolled parents: most (85%) were female and identified as mothers (78%), sixty percent self-identified as White, 38% as Black, and 63% had graduated from college or had post-graduate education. Half were married and slightly over half reported economic perception of poor, almost poor, or living check-to-check. The average age of their child was 12.5 years (± 2.7yrs) and half were female.

**Table 1:** Parent characteristics.

Variables	Responses	*N* (%) or Mean (SD)
Parent/Legal Guardian sex	Female	34 (85)
	Male	6 (15)
Parent race	More than 1	1 (3)
	Black	15 (38)
	White	24 (60)
Economic perception*	Less well	21 (53)
	Comfortable	19 (48)
Parental Education	HS to some college	15 (38)
	College or more	25 (63)
Marital Status	Not married	20 (50)
	Married	20 (50)
Child age	8-12 years	20 (50)
	13-17 years	20 (50)
Child sex	Female	20 (50)
	Male	20 (50)
Child race	More than 1	7 (18)
	Black	15 (38)
	White	18 (45)
Asthma Severity	Mild	9 (23)
	Moderate	15 (38)
	Severe	16 (40)
Exposure to second-hand tobacco smoke	Yes	11 (28)
	No	29 (73)
Forced expiratory volume in 1 sec (FEV1)	Percent Predicted	102 (14)
FEV1/Forced Vital Capacity (FVC) ratio	Percent Predicted	94.6 (7.0)
Positive skin test and/or serum specific IgE ≥0.35 IU/ml to at least 1 aeroallergen	Yes	21 (53)
	No	7 (18)
	Unknown	12 (30)
Emergency Department visit for asthma exacerbation in past 12 months	Yes	19 (48)
	No	21 (53)

Percentages may be > 100 from rounding.

*Category “less well” includes poor, almost poor, and living from check to check; “comfortable” includes living comfortably and living very well. Race, ethnicity, and sex were self-reported. Severity was determined by therapy per NAEPP EPR3 guidelines: step 2- mild, step 3 / 4- moderate, step 5 / 6- severe. Children receiving a biologic immunomodulator were categorized as step 5. Percent predicted uses Global Lung Initiative (GLI) 2012 prediction equations.

Table 2 displays parental responses by category: awareness, perception, behavior change, and discussion with healthcare providers. Almost all parents reported awareness of the AQI or alerts, with only 2 (5%) reporting no knowledge. Despite such awareness, only one-fifth of parents reported checking the AQI by the government-sponsored AirNow app or website. Two-thirds of parents reported outdoor air pollution as a trigger for their child's asthma, but less than one-quarter reported air pollution caused an illness or symptom in the prior year. Most parents reported they did not alter their child's outdoor activity in the last year based on perceived air quality (58%), or on the AQI or alerts (60%). Half of all parents did not instruct their child to avoid busy roads when walking, biking, or exercising to reduce exposure to air pollution, or practice these avoidance behaviors themselves. Slightly over half of parents reported ever discussing outdoor air pollution as a possible trigger for their child's asthma with healthcare providers, with only 23% of parents reporting healthcare provider instruction to reduce or change their child's activity in response to poor air quality. Most parents wanted such discussion with healthcare providers.

**Table 2:** Parental knowledge and usage of the Air Quality Index (AQI) in parents of children with persistent asthma.

Questions	Responses	*N* (%)
**Air Quality Awareness**		
Have you ever heard or read about the air quality index or air quality alerts where you live?	Yes	38 (95%)
	No	2 (5%)
Past 12 months: Have you ever checked the air quality index on the smart phone app (AIRNOW) or on the website (airnow.gov)?	Yes	8 (20%)
	No	32 (80%)
**Perception of Air Quality and Health**		
Do you think outdoor air pollution is a trigger for your child’s asthma?	Yes	26 (65%)
	No	14 (35%)
Past 12 months: Has your child had an illness or symptom that you think was cause by pollution in the air outdoors?	Yes	7 (18%)
	No	33 (83%)
**Behavioral Modification in Response to Air Quality**		
Past 12 months: Reduced or changed child’s activity level because the air quality was bad or was affecting how child felt?	Yes	17 (43%)
	No	23 (58%)
Past 12 months: # times reduced or changed child's outdoor activity level based on the air quality index or air quality alerts?	0 times	24 (60%)
	≥2 times	16 (40%)
How often do you tell your child to avoid busy roads to reduce exposure to air pollution when walking, biking, or exercising outdoors?	None of the time	21 (53%)
	A little of the time	10 (25%)
	Some of the time	4 (10%)
	Most of the time	5 (13%)
	All of the time	0 (0%)
How often do you avoid busy roads to reduce exposure to air pollution when walking, biking, or exercising outdoors?	None of the time	20 (50%)
	A little of the time	7 (18%)
	Some of the time	7 (18%)
	Most of the time	5 (13%)
	All of the time	1 (23%)
**Health Care Provider Discussion of Air Quality**		
Doctor, nurse of health professional ever talked about outdoor air pollution as an asthma trigger?	Yes	21 (53%)
	No	19 (48%)
Doctor, nurse, or health professional recommended outdoor activity change when air quality is bad?	Yes	9 (23%)
	No	31 (78%)
Want your child's doctor or healthcare professional to discuss outdoor air pollution?	Yes	22 (55%)
	No	18 (45%)

Values may be greater than 100% due to rounding.

Table 3 shows the results of the analysis of parental behavior change by selected variables. Parents who changed their child's behavior due to perceived air quality (i.e., thought air quality was bad or affecting their child), were more likely to perceive air pollution as an asthma trigger (88%) and report discussion of air pollution as a possible trigger with a healthcare provider (76%). For parents who did not change behavior based on perception of poor air quality, most did not endorse air pollution as an asthma trigger (52%), cause of illness or symptom in the prior 12 months (96%), report discussion with healthcare provider (65%) including recommendation for behavior change in response to poor air quality (91%), or usage of the AQI *via* AirNow (96%).

**Table 3:** Analysis of select variables and reported outdoor activity modifications based on air quality in children with persistent asthma.

	Reduced or changed child’s activity level because the air quality was bad or affecting how well child felt				Number times reduced or changed child's outdoor activity level based on the AQI or air quality alerts		
Variables	Responses	**Yes**	**No**	***P***	≥**2 times**	**0 times**	***P***
Perception of air pollution as an asthma trigger	Yes	15 (88)	11 (48)	0.02	13 (81)	13 (54)	0.10
	No	2 (12)	12 (52)		3 (19)	11 (46)	
Thought child had air pollution illness in last 12 months	Yes	6 (35)	1 (4)	0.03	7 (44)	0 (0)	< 0.01
	No	11 (65)	22 (96)		9 (56)	24 (100)	
Health professional discussion of outdoor air pollution as asthma trigger	Yes	13 (76)	8 (35)	0.01	11 (69)	10 (42)	0.12
	No	4 (24)	15 (65)		5 (31)	14 (58)	
Health professional recommendation to reduce or change outdoor activity level when bad air quality	Yes	7 (41)	2 (9)	0.02	7 (44)	2 (8)	0.02
	No	10 (59)	21 (91)		9 (56)	22 (92)	
Checked the air quality index on the smart phone app (AIRNOW) or on the website (airnow.gov)	Yes	7 (41)	1 (4)	< 0.01	8 (50)	0 (0)	< 0.01
	No	10 (59)	22 (96)		8 (50)	24 (100)	

*N* (%) displayed as column percentages. Percentages may be > 100 from rounding. *P* value obtained from Fischer’s exact test.

Parents endorsing at least one behavioral change based on AQI or air quality alerts were more likely to perceive air pollution as an asthma trigger (81%) and report healthcare provider discussion of air pollution as a trigger (69%), though this was not statistically significant. Half of all parents who endorsed at least one behavioral change reported checking the AQI on AirNow. Of the parents who reported no behavior change in the last 12 months based on the AQI or air quality alerts, none of the parents thought air pollution caused an illness or symptom, most did not have healthcare provider discussions (58%) or recommendations for behavioral change (92%), and no parent reported checking the AQI on AirNow smartphone app or website. All parents who checked the AirNow app or website reported at least one behavioral change based on AQI or alert in the prior year (*p* < 0.01).

Behavioral changes in response to perceived air quality or based on alerts/AQI did not differ by parental or child sex, age, race, nor by parental education, economic perception, or marital status (Supplementary Materials Table S2**)**.

## Discussion

Our study focuses on parental awareness of the AQI and usage of such in their child's asthma management, one of only two such studies in the US (6) and the first study to assess usage of AirNow as a source of AQI information. Awareness of the AQI or air quality alerts was nearly universal among parents of children with persistent asthma, however relatively few parents endorsed behavioral modification to reduce air pollution exposure.

Our findings are consistent with McDermott et al., who reported 88% of parents of children with and without asthma were aware of air quality alerts, although such knowledge was not associated with anticipated behavioral changes based on the number of poor air quality days (6). As a prior study of US adults found awareness of air quality alerts and behavioral change was increased in areas with more alerts (issued when AQI ≥101) (9), it is possible our findings are similar to McDermott's given both regions have poor air quality (13). Such, it is unknown if our high percentage of parents reporting knowledge of alerts translates to parents of children with asthma residing in areas with improved or differing air quality. Regardless of region, we speculate most parents of children with asthma will have greater knowledge of alerts given national studies in adults which consistently report increased air quality alert awareness in persons with asthma (8, 14) or respiratory disease (15). Our results agree with prior studies in that persons with respiratory disease more frequently report behavioral modifications in response to poor air quality, yet the overall percentages of those reporting changes are low (8, 9, 16).

We found that despite awareness of air quality alerts, only a small percentage of parents checked the AQI on the EPA's AirNow smartphone app or website. A 2014 study found that most adults received their air alerts *via* television (17), although it is unclear if this holds true today given the wide-spread availability of smartphones and apps. A more recent study found adolescents with asthma most often heard or read about air quality alerts on mobile apps (18). An important consideration of air alerts is that they are often issued on days when the AQI is forecast to be at the orange level and above (≥101), yet adverse childhood asthma outcomes have been reported for concentrations below that level (19, 20). Such, it is unclear how many children with asthma should follow behavioral modification advice for the AQI moderate category (51–100), which states “… pollution in this range may pose a moderate health concern for a very small number of individuals. People who are unusually sensitive to ozone or participle pollution may experience respiratory symptoms.” (21). Thus, for the AQI to be used as a tool for parents and children to learn individual susceptibilities to air pollution, the AQI must be used outside of just alerts. Therefore, parents and children must be able to find and check current AQI information (i.e., have knowledge of AirNow). Our findings of a low percentage of parents checking AirNow coupled with increased behavioral change amongst those who do, support AirNow education during clinical asthma management encounters.

Unlike other asthma triggers, air pollution's physical characteristics (e.g., colorless, odorless) and delayed onset of symptoms (i.e., lag times) may make it difficult for patients, parents, and health care providers to identify it as an asthma trigger. We found the majority of parents reported air pollution as a trigger for their child's asthma, which is higher than a prior study in which only 3% of parents reported such (22). The difference could represent selection bias or residence in a region with increased air quality awareness. Perception of air pollution as a trigger or cause of illness was higher amongst parents reporting outdoor activity modification. Future studies are needed examining if parental perception of air pollution as a trigger for their child's asthma is associated with increased outdoor air pollution susceptibility.

Consistent with prior studies in adolescents (23, 24), we found that parents of children with asthma did not endorse self nor child avoidance of activity near busy roadways. Given the known harms associated with traffic-related air pollution (TRAP) exposure for persons with asthma, the US Environmental Protection Agency advises avoidance of busy roadways (25). Our results and those of others support the need for healthcare provider discussions regarding the harms of activity near roadways. Such discussions will undoubtedly be influenced by structural and social determinants of health and should consider a child's access to safe activity areas with reduced air pollution, temporality of air pollution (i.e., TRAP highest during rush hours), and the numerous benefits of physical activity (26).

Although half of parents reported a prior healthcare provider discussion about air pollution as an asthma trigger, less than a quarter of parents reported receiving behavioral avoidance recommendations. Compared to existing studies, our results are consistent with a national study noting slightly over half (56%) of pediatricians reported patient discussions about air quality, although that study was limited by lack of specification of indoor verses outdoor pollution (27). Conversely, in studies of US adolescents only 3% overall (23) and 12% with asthma (18) reported discussion of air pollution with a healthcare provider. An important consideration is that our sample was recruited from children predominately seeking asthma management from specialists. Thus, the higher percentage of parents reporting discussion may be biased by our selection. However, if our sample is biased by parents more likely to have discussed air pollution with a healthcare provider, it is further concerning that less than a quarter of parents reported receiving outdoor activity modification recommendations in response to poor air quality. This is particularly important given our results are consistent with national studies noting associations between healthcare provider discussion and increased behavioral modification (8, 14).

Our finding that only half of parents reported a healthcare provider discussion regarding outdoor air pollution as an asthma trigger requires further investigation. The National Asthma Education and Prevention Program guidelines recommend discussion of air quality during asthma care (2) although the recommendations are less specific compared to other well-known environmental triggers. For example, the most recent update (Expert Panel Review 4) provides clear clinical recommendations regarding indoor aeroallergen management (28). The factors contributing to reduced provider discussions are unknown but could include lack of detailed national asthma guideline recommendations, inadequate knowledge of the harms of outdoor air pollution, lack of evidence demonstrating improvement in asthma outcomes when following AQI recommendations, concern for inability to change outdoor air pollution exposure for patients, and/or preferential targeting of indoor asthma triggers such as aeroallergens and tobacco smoke exposure.

Notably, there have been no large prospective studies of the association of the AQI and childhood asthma health outcomes. While our prior work demonstrated an association of the AQI and childhood asthma emergency department visits (20), primarily driven by fine particle pollution, it is unknown if the AQI is predictive of adverse childhood asthma outcomes in all regions nor if adherence to the AQI and its recommendations can prevent adverse asthma outcomes. As the AQI represents the highest individual pollutant and does not consider the effects of multiple pollutants, the health impacts associated with AQI could differ regionally and by varying pollutant concentrations. For example, a recent study in California found the AQI was associated with all cause adult respiratory emergency department visits when and where the AQI strongly correlated with fine particulate matter (29). Given the well-established link between outdoor air pollution and asthma exacerbations in children, it remains prudent to recommend usage of the AQI and speculatively, it is likely to be most beneficial when used as a tool to learn personal susceptibilities.

We acknowledge our study is limited by a small sample size, however our findings agree with results from national studies. Additionally, our study is limited by the recruitment of parents in whom most children were receiving asthma specialist care in a region with high air pollution levels. Thus, our findings may not be generalizable to all children with asthma, rural locations, or regions with improved air quality. Additionally, we did not ask about other sources of air quality information (e.g., PurpleAir, Breezometer, etc) and although mostly adapted from existing questions, our survey was not validated. Validation of our survey is needed prior to future studies. Lastly, we did not assess knowledge of indoor air quality or interventions, which can also be an important source of air pollution exposure.

When discussing behavioral interventions for outdoor air pollution, such as the Air Quality Index, it is important to note that the most equitable and impactful interventions are those at a policy level (30). Indeed, the United States (US) has experienced marked reductions in outdoor air pollution as a result of the Clean Air Act. Unfortunately, reductions are often inequitably experienced, and in general wealthier non-Hispanic White persons benefit from lower outdoor air pollutant exposure compared to other groups (31). For example, more non-US born, under-resourced, and Black and Hispanic persons live closer to major roadways compared to non-Hispanic White persons (32). Thus, personal interventions are likely to widen inequities by placing the burden of air pollution on individuals, thus a matter of environmental justice (30). However, the preponderance of evidence supports outdoor air pollution harms children with asthma and healthcare providers should discuss this as part of management. Such discussions should include assessment of potential risks, benefits, and harms (e.g., anxiety, sedentarism) of behavioral interventions (30). For example, if avoidance of exercise near a major roadway is not possible, times when pollution is likely to be lower (e.g., non-rush hours times) and the importance of physical activity for overall health could be discussed (33, 34).

In conclusion, our findings suggest that healthcare providers, as part of asthma management in children, should discuss outdoor air pollution as an asthma trigger while also recommending usage of the AirNow (app or website) to guide appropriate behavioral avoidance measures. Future studies of large cohorts of children living in different geographic regions are needed to confirm our study findings as well as assess the association and efficacy of AQI usage on improving asthma outcomes.

## Data Availability

The original contributions presented in the study are included in the article/Supplementary materials, further inquiries can be directed to the corresponding author/s.
