# Supplementary material for: Parental knowledge and usage of air quality in childhood asthma management
Source: Front Pediatr. 2022 Oct 26;10:966372. doi: 10.3389/fped.2022.966372 (PMC9687089; doi:10.3389/fped.2022.966372)
Supplement: Supplementary file 1 [file Table1.docx]

| Table S1: Questionnaire administered to parents | | | | | | | | | |
| --- | --- | --- | --- | --- | --- | --- | --- | --- | --- |
| **Air Quality Awareness** | | | | | | | | | |
| 1. The government routinely collects information on air quality that may be distributed by local radio, TV and newspapers to help inform the public about air pollution levels.  Have you ever heard or read about the air quality index or air quality alerts where you live? | | | | | | | | | |
| Yes | | | No | | | | Don’t know/Not sure | | |
| 1. Please think of the past 12 months. Have you ever checked the air quality index on the smart phone app (AIRNOW) or on the website (airnow.gov)? | | | | | | | | | |
| Yes | | | No | | | | Both | | |
| **Perception of Air Quality and Health** | | | | | | | | | |
| 1. Children with asthma have many different triggers for their asthma. For example, some children have more asthma symptoms such as coughing or chest tightness when they get sick with a cold. Do you think outdoor air pollution is a trigger for your child’s asthma? | | | | | | | | | |
| Yes | No | | | | Don’t know/Not sure | | | I’ve never thought about it | |
| 1. Things like smog, automobile exhaust, and chemicals can cause outdoor air pollution. In the past 12 months has your child had an illness or symptom that you think was cause by pollution in the air outdoors?   Note: this question does not refer to natural agents like pollen or dust in outdoor air | | | | | | | | | |
| Yes | | | No | | | | Don’t know/Not sure | | |
| **Behavioral Modification in Response to Air Quality** | | | | | | | | | |
| 1. Please think about the past 12 months. Did you reduce or change your child’s activity level because you thought the air quality was bad or was affecting how well your child felt? | | | | | | | | | |
| Yes | | | No | | | | Don’t know/Not sure | | |
| 1. Please think of the past 12 months. How many times did you reduce or change your child's outdoor activity level based on the air quality index or air quality alerts?   ____________  Enter zero if no reduction or change because of air quality index or alerts | | | | | | | | | |
| 1. How often do you tell your child to avoid busy roads to reduce exposure to air pollution when walking, biking, or exercising outdoors? | | | | | | | | | |
| None of the time | | A little of the time | | Some of the time | | Most of the time | | | All of the time |
| 1. How often do you avoid busy roads to reduce exposure to air pollution when walking, biking, or exercising outdoors? | | | | | | | | | |
| None of the time | | A little of the time | | Some of the time | | Most of the time | | | All of the time |
| **Health Care Provider Discussion of Air Quality** | | | | | | | | | |
| 1. Has a doctor, nurse of health professional ever talked to you about outdoor air pollution as being a possible trigger for child's asthma? | | | | | | | | | |
| Yes | | | No | | | | Don’t know/Not sure | | |
| 1. Has a doctor, nurse, or health professional ever told you to reduce or change your child's outdoor activity level when the air quality is bad? | | | | | | | | | |
| Yes | | | No | | | | Don’t know/Not sure | | |
| 1. Do you want your child's doctor or healthcare professional to talk to you about outdoor air pollution? | | | | | | | | | |
| Yes | | | No | | | | Don’t know/Not sure | | |
| Question order differed on REDcap questionnaire. | | | | | | | | | |

**Sources of questions**:

**1**. From the Behavioral Risk Factor Surveillance System (BRFSS) 2005 and ConsumerStyles surveys.

Question: “ **The government routinely collects information on air quality that may be distributed by local radio, TV and newspapers to help inform the public about air pollution levels. Have you ever heard or read about the air quality index or air quality alerts where you live?**”

Prior published results: **1.** Wen XJ, Balluz L, Mokdad A. Association between media alerts of air quality index and change of outdoor activity among adult asthma in six states, BRFSS, 2005. J Community Health. 2009;34(1):40-6. **2.** Mirabelli MC, Ebelt S, Damon SA. Air Quality Index and air quality awareness among adults in the United States. Environ Res. 2020;183:109185.

**2.** Novel question.

**3**. Novel question.

**4**. Adapted from Behavioral Risk Factor Surveillance System (BRFSS) 2004.

Original Question: “**Things like smog, automobile exhaust, and chemicals can cause outdoor air pollution. In the past 12 months, have you had an illness or symptom that you think was caused by pollution in the air outdoors? Note: this question does not refer to natural agents like pollen or dust in outdoor air**.“

Available at: <https://www.cohealthdata.dphe.state.co.us/chd/Resources/brfss/FinalCO04.pdf>, accessed 09/01/2022.

**5**. Adapted from Behavioral Risk Factor Surveillance System (BRFSS) 2005.

Original Question: “ **Please think of the past 12 months. How many times did you reduce or change your outdoor activity level because you thought the air quality was bad or was affecting how well you felt**?”

Prior published results: Wen XJ, Balluz L, Mokdad A. Association between media alerts of air quality index and change of outdoor activity among adult asthma in six states, BRFSS, 2005. J Community Health. 2009;34(1):40-6.

Adapted from National Health and Nutrition Examination Survey (NHANES) 2009-2010, data file AQQ_F.xpt., Air Quality (AQQ_F) and from question #6.

Original Question: “**During the past 12 months, when {you thought/SP though} or {were /was} informed air quality was bad, {did you/did SP} do anything differently**?

Answer choices: Yes, No, SP never thought/informed bad air quality, refused, don’t know, missing.

Prior published results: Wells EM, Dearborn DG, Jackson LW. Activity change in response to bad air quality, National Health and Nutrition Examination Survey, 2007-2010. PloS one. 2012;7(11):e50526.

**6**. Adapted from Behavioral Risk Factor Surveillance System (BRFSS) 2005.

Original Question: “ **Please think of the past 12 months. How many times did you reduce or change your outdoor activity level based on the air quality index or air quality alerts?**”

Prior published results: Wen XJ, Balluz L, Mokdad A. Association between media alerts of air quality index and change of outdoor activity among adult asthma in six states, BRFSS, 2005. J Community Health. 2009;34(1):40-6.

**7**. Adapted from ConsumerStyles survey, 2014-2016.

Original Question: “**When walking, biking, or exercising outdoors, how often do you avoid busy roads to reduce your exposure to air pollution?**”

Answer choices: Always, usually, sometimes, rarely, never, don’t know.

Prior published results: **1.** Mirabelli MC, Boehmer TK, Damon SA, Sircar KD, Wall HK, Yip FY, et al. Air Quality Awareness Among U.S. Adults With Respiratory and Heart Disease. Am J Prev Med. 2018;54(5):679-87. **2.** Lynch KM, Mirabelli MC. Air Quality Awareness and Behaviors of U.S. Adolescents With and Without Asthma. Am J Prev Med. 2021

**8**. Adapted from ConsumerStyles survey, 2014-2016.

Original Question: “**When walking, biking, or exercising outdoors, how often do you avoid busy roads to reduce your exposure to air pollution?**”

Answer choices: Always, usually, sometimes, rarely, never, don’t know.

Prior published results: **1.** Mirabelli MC, Boehmer TK, Damon SA, Sircar KD, Wall HK, Yip FY, et al. Air Quality Awareness Among U.S. Adults With Respiratory and Heart Disease. Am J Prev Med. 2018;54(5):679-87. **2.** Lynch KM, Mirabelli MC. Air Quality Awareness and Behaviors of U.S. Adolescents With and Without Asthma. Am J Prev Med. 2021

**9**. Novel question with influence from question 10.

**10.** Adapted from Behavioral Risk Factor Surveillance System (BRFSS) 2005.

Original Question: “**Has a doctor, nurse, or other health professional ever told you to reduce your outdoor activity level when the air quality is bad?**”

Prior published results: Wen XJ, Balluz L, Mokdad A. Association between media alerts of air quality index and change of outdoor activity among adult asthma in six states, BRFSS, 2005. J Community Health. 2009;34(1):40-6.

**11**. Novel question.

| Table S2: Analysis of select variables and outdoor activity modifications based on air quality in children with persistent asthma | | | | | | | | |
| --- | --- | --- | --- | --- | --- | --- | --- | --- |
|  | | | Reduced or changed child’s activity level because the air quality was bad or affecting how well child felt | | | Number times reduced or changed child's outdoor activity level based on the AQI or air quality alerts | | |
| Variables | | Responses | **Yes** | **No** | **P** | $\boldsymbol{\geq}$**2 times** | **0 times** | **P** |
| Parental/Legal Guardian sex | | Female  Male | 15 (88)  2 (12) | 19 (83)  4(17) | 1.00 | 14 (88)  2 (13) | 20 (83)  4 (17) | 1.00 |
| Parental race | | $>1$  Black  White | 1 (4)  7 (30)  15 (65) | 0 (0)  8 (47)  9 (53) | 0.61 | 0 (0)  6 (38)  10 (63) | 1 (4)  9 (38)  14 (58) | 1.00 |
| Economic perception* | | Less well  Comfortable | 9 (53)  8(47) | 12 (52)  11 (48) | 1.00 | 8 (50)  8(50) | 13 (54)  11(46) | 1.00 |
| Parental Education | HS to some college  College or more | | 9 (53)  8 (47) | 6 (26)  17 (74) | 0.11 | 8 (50)  8 (50) | 7 (29)  17 (71) | 0.20 |
| Marital Status | | Married  Not married | 6 (35)  11 (65) | 14 (61)  9 (39) | 0.20 | 13 (54)  11 (46) | 7 (44)  9 (56) | 0.75 |
| Child age | | 8-12 years  13-17 years | 11 (65)  6 (35) | 9 (39)  14 (61) | 0.20 | 9 (56)  7 (44) | 11 (46)  13 (54) | 0.75 |
| Child sex | | Female  Male | 10 (59)  7 (41) | 10 (43)  13 (57) | 0.52 | 11 (69)  5 (31) | 9 (38)  15 (63) | 0.11 |
| Child race | | $>1$  Black  White | 3 (18)  8 (47)  6 (35) | 4 (17)  7 (47)  12 (52) | 0.49 | 4 (25)  5 (31)  7 (44) | 3 (13)  10 (42)  11 (46) | 0.63 |
| N (%) are displayed as column percentages. Percentages may be > 100 from rounding. P value obtained from Fischer’s exact test. Category “less well” includes poor, almost poor, and living from check to check; “comfortable” includes living comfortably and living very well. | | | | | | | | |
